# Pharmacogenetic Analysis of TPMT and NUDT15 in a European Pediatric Cohort with IBD and Autoimmune Diseases: Frequency Data and Clinical Relevance

**DOI:** 10.3390/genes16111372

**Published:** 2025-11-11

**Authors:** Anna Pau, Ilaria Galliano, Alice Ponte, Anna Clemente, Maddalena Dini, Cristina Calvi, Paola Montanari, Antonio Pizzol, Stefano Gambarino, Pier Luigi Calvo, Massimiliano Bergallo

**Affiliations:** 1 Department of Public Health and Pediatrics, School of Medicine, University of Turin, 10126 Turin, Italy; anna.clemente@unito.it (A.C.); maddalena.dini@unito.it (M.D.); cristina.calvi@unito.it (C.C.); paola.montanari@unito.it (P.M.); stefano.gambarino@unito.it (S.G.); massimiliano.bergallo@unito.it (M.B.); 2 Laboratory of Specialistic Pediatry, Department of Pediatric Pathology and Care, Regina Margherita Children’s Hospital, 10126 Turin, Italy; 3 Unit of Pediatric Gastroenterology, Department of Pediatric Pathology and Care, Regina Margherita Children’s Hospital, 10126 Turin, Italy; pontealice236@gmail.com (A.P.); apizzol@cittadellasalute.to.it (A.P.); pcalvo@cittadellasalute.to.it (P.L.C.)

**Keywords:** *TPMT*, *NUDT15*, thiopurine, pharmacogenetic, IBD, pediatrics

## Abstract

Background/Objectives: Thiopurines remain a cornerstone in the management of inflammatory bowel disease (IBD) and gastrointestinal immune diseases but are associated with significant interindividual variability in efficacy and toxicity, mainly influenced by polymorphisms in Thiopurine S-methyltransferase *TPMT* and Nudix Hydrolase 15 *NUDT15.* This study aimed to assess the frequency of *TPMT* and *NUDT15* variants in a pediatric cohort and evaluate their clinical impact to support a pharmacogenetic-guided approach to thiopurine therapy. Methods: Eighty-three pediatric patients with IBD and other autoimmune diseases were genotyped for clinically relevant *TPMT* and *NUDT15* variants using two HRM-PCR assays and were confirmed with sequencing. Variant frequencies were compared to expected population data, and clinical records were reviewed to assess thiopurine dosing, tolerance, and adverse events. Results: Among the cohort, six carried heterozygous *TPMT* variants *1/*3A, while 2 carried the *NUDT15* *1/*9 diplotype, with frequencies higher than expected. Among patients with *TPMT* variant alleles, some needed dose reductions or treatment discontinuation due to adverse effects, while others tolerated standard dosing without significant issues. Notably, no significant differences in adverse reactions were observed between *NUDT15* *1/*9 carriers and wild-type patients. Conclusions: Our results confirm the clinical relevance of *TPMT* and *NUDT15* genotyping to personalize thiopurine therapy in pediatric IBD. Routine implementation of rapid genetic testing, combined with therapeutic drug monitoring and a structured management algorithm, may optimize treatment outcomes and minimize preventable toxicity.

## 1. Introduction

The era of personalized medicine aims to optimize therapeutic efficacy and minimize toxicity by tailoring treatment to individual patients. Cytotoxic drugs often show substantial inter-individual variability, and pharmacogenetics provides a key strategy to mitigate this risk [1]. In inflammatory bowel disease (IBD), thiopurines such as mercaptopurine (MP) and 6-thioguanine (TG) are widely used immunosuppressants, but patient response and toxicity vary markedly due to genetic differences in drug metabolism. Pre-treatment genotyping of drug-metabolizing enzymes allows for identification of allele variant carriers and guides dosing adjustments, a practice strongly recommended by international consortia for various chemotherapies, including thiopurines [2].

**Figure 1:**
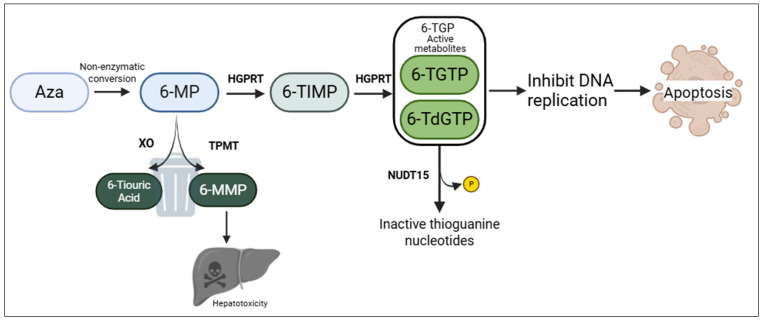
Metabolic pathway of azathioprine.

The thiopurines mercaptopurine (MP) and 6-thioguanine (TG) were developed in the 1950s by Elion and Hitchings. Initially used as antitumour agents, they were later employed in the treatment of immune-mediated diseases such as IBD and rheumatoid arthritis. Despite the widespread use of TNF antagonists, thiopurines remain key co-immunosuppressants to prevent anti-drug antibodies and improve clinical response [3,4]. The metabolic pathway of azathioprine is shown in Figure 1. Bioavailability of MP is increased by the conjugation of the imidazole moiety, forming the prodrug azathioprine (AZA), converted to 6-mercaptopurine (6-MP).

6-MP is then primarily metabolized by two enzymes: thiopurine S-methyltransferase (*TPMT*) and xanthine oxidase (XO), which convert it, respectively, into 6-thiouric acid (6-TUA) and 6-methylmercaptopurine (6-MMP). Elevated levels of 6-MMP in erythrocytes are associated with an increased risk of liver injury [5,6,7]. Active metabolites, the 6-thioguanine nucleotides (6-TGN), accumulate in red blood cells, and their concentrations increase following inhibition of enzymes involved in AZA metabolism, thereby enhancing immunosuppressive efficacy [8,9,10]. 6-TGN exerts pharmacological activity in the form of 6-thio-(deoxy) guanosine-triphosphate (6-T(d)GTP), which is incorporated into DNA or RNA as a guanosine analogue, blocking nucleic acid synthesis and lymphocyte proliferation [11].

A genetic variant of the Thiopurine S-methyltransferase *TPMT* gene may be identified before starting therapy and may consequently be treated with an alternative therapy or with a markedly reduced drug dosage. The allelic variants included in this study (*TPMT* *2, *3A, *3B, *3C; *NUDT15* *3) correspond to those recommended in the AMP minimum testing guidelines (tier 1) and are consistent with NCCN recommendations for thiopurine toxicity risk assessment [12,13].

**Figure 2:**
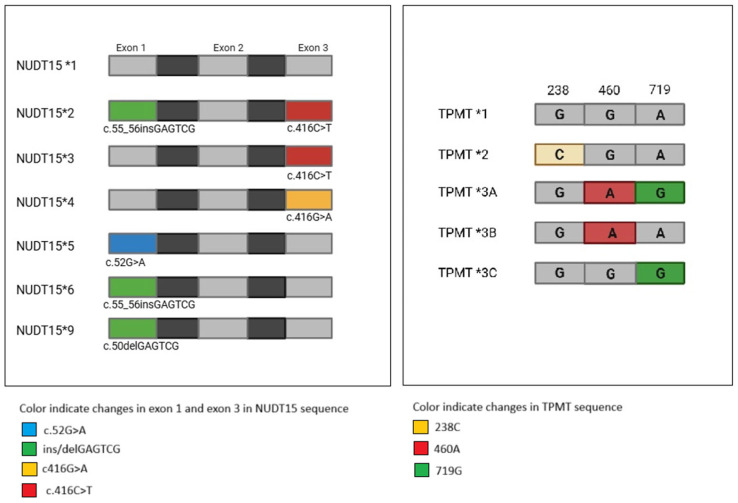
Visual representation of *NUDT15* and *TPMT* mutations defining variant alleles.

Guidelines recommend reducing the starting dose for *TPMT* intermediate metabolizers (IMs) to 30–70% of the standard dose, and for poor metabolizers (PMs) either administering a tenfold reduced dose three times per week or considering an alternative medication. It is known that patients with no or reduced activity of the enzyme thiopurine S-methyltransferase (*TPMT*) have an increased risk of hematological toxicity, hepatic toxicity under the therapy with 6-thioguanine, 6-mercaptopurine, or azathioprine [14,15,16]. Genetic modifications of *TPMT* may cause an amino acid exchange. The resulting altered conformation of the enzyme has an influence on its activity. The human *TPMT* gene is genetically polymorphic, with 90% of Caucasians carrying wild-type alleles with high *TPMT* activity, 10% exhibiting intermediate *TPMT* activity, and 0.3% displaying low *TPMT* activity [17]. The *TPMT* gene’s wild-type allele, referred to as *1, contrasts with several variant alleles, including TPMT*3A (harboring c.460G>A, rs1800460 and c.719A>G, rs1142345), TPMT*3B (c.460G>A, rs1800460), TPMT*3C (c.719A>G, rs1142345) and TPMT*2 (c.238G>C, rs1800462). All the variant alleles are represented in Figure 2. These are the most frequent *TPMT* alleles, accounting for 80–95% of intermediate or low enzyme activity across different populations worldwide [18]. Comparative investigations of genotype and phenotype observed a correlation of 87% between genotype and enzyme activity. Severe and sometimes fatal myelosuppression can occur in patients treated with thiopurine drugs who have complete *TPMT* deficiency due to homozygous or compound heterozygous TPMT*3A, TPMT*3B, TPMT*3C, or TPMT*2 genotypes [3]. In four patients with complete *TPMT* deficiency, the time between starting thiopurine drug therapy and experiencing bone marrow toxicity was less than 1.5 months [19]. Likewise, cases of severe or fatal pancytopenia have been reported in patients with homozygous *TPMT* genotypes and complete enzyme deficiency, with myelotoxicity emerging three to seven weeks after therapy initiation [3]. One third of *TPMT* wild-type patients treated for IBD experience thiopurine toxicity, which is a high incidence in Asian patients [20]. However, variant *TPMT* genotypes are less common in Southwest Asians (2.0%), Chinese (4.7%), and Japanese (2.0%) populations than in Caucasian populations, where the genotype frequency is 10.1% [3].

Alongside *TPMT* polymorphisms, Nudix Hydrolase 15 *NUDT15* is a key target in pharmacogenetic studies evaluating inter-individual differences in thiopurine response. Genetic polymorphisms affecting the enzyme *NUDT15* have been linked to severe thiopurine-induced toxicity, particularly in Asian patients undergoing thiopurine therapy, providing a potential explanation for the pharmacological activity of 6-TGN [21]. *NUDT15* hydrolyzes cytotoxic thioguanine triphosphates (TGTP) to their monophosphate form, preventing their incorporation into DNA [22]. Knockdown of *NUDT15* in a human lymphoid cell line was associated with significantly increased levels of TGTP and TdGTP, as well as higher levels of thioguanine in the DNA. This resulted in increased apoptosis [23]. Patients carrying *NUDT15* loss-of-function variants experience thiopurine-induced cytotoxicity, such as myelosuppression and alopecia. The *NUDT15* gene has been found to contain several clinically relevant variants [Figure 2], including two affecting residue Arg139 in exon 1: c.415C>T (p.Arg139Cys) and c.416G>A (p.Arg139His). Additionally, two variants affect the Val18 in exon 3: c.52G>A (p.Val18Ile) and the insertion variant c.36_37insGGAGTC (p.Val18_Val19insGlyVal). The combination of these variants defines five distinct haplotypes: NUDT15*1 represents the wild-type allele; p.Arg139Cys defines NUDT15*3 (rs116855232); the insertion p.Val18_Val19insGlyVal corresponds to NUDT15*6 (rs869320766); NUDT15*2 combines p.Arg139Cys and p.Val18_Val19insGlyVal; p.Arg139His defines NUDT15*4; p.Val18Ile defines NUDT15*5 (rs186364861); and the deletion p.G17_V18del (rs746071566, delGGAGTC) defines NUDT15*9 [3,24]. The frequency of *NUDT15* variants varies by ethnicity, as reported by the Pharmacogene Variation Consortium (PharmVar): for example, the p.Arg139Cys allele (NUDT15*2 and 3) is common in Asian populations but has also been reported in Hispanic patients, whereas NUDT15*9 has been observed only in European populations [21]. In Korean Crohn’s disease patients [25], the *NUDT15* c.415C>T variant strongly predicts severe thiopurine-induced myelotoxicity, outperforming *TPMT* variants (sensitivity 89.4% vs. 12.1%, specificity 93.2% vs. 97.6%). Its allele frequency is 13% in Chinese, 7% in Japanese, 10% in Koreans, and 2% in admixed Americans. *TPMT* haplotypes *2 and *3 were classified as a complete loss of function in functional studies [23]. According to the Clinical Pharmacogenetics Implementation Consortium (CPIC), patients who carry both alleles are considered to be poor metabolizers, while those who carry one functional allele (either *1/2 or 1/3) are classified as intermediate metabolizers [2].

In vitro studies of NUDT15 *5 and *6 indicate a 50–60% reduction in enzymatic activity, but their in vivo significance remains unclear [23]. A previous study reported lower diagnostic accuracy for NUDT15 *5 and *6 compared to NUDT15*3 (Cargnin et al.), and therefore patients carrying one non-functional allele and one variant of uncertain significance may be classified as possible intermediate metabolizers [2]. Variants *4 and 9 are rare; they show reduced *NUDT15* activity in vitro but have limited evidence of clinical relevance for thiopurine toxicity and are therefore considered to have uncertain function.

Genome-wide association studies (GWAS) show *TPMT* variants mainly affect thiopurine tolerance in European and African patients, while *NUDT15* is key in Asian patients. Consequently, the FDA and EMA updated mercaptopurine labeling to highlight dose reductions for *NUDT15*-defective carriers. Clinical guidelines, including CPIC, recommend pre-treatment *TPMT* and *NUDT15* genotyping to ensure safe dosing [2].

Genetic testing before thiopurine therapy is increasingly used and shown to be cost-effective in reducing toxicity and improving safety; however, it is not yet routine in all settings. Costs vary by method, with sequencing still more expensive and less accessible than targeted genotyping for key *TPMT* and *NUDT15* variants.

However, this practice limits our understanding of the true impact of pharmacogenetics and the prevalence of relevant mutations. With this study, we aim to explore the clinical implications of thiopurine pharmacogenetics by analyzing the frequency of variants in a predominantly Caucasian pediatric population and by investigating differences in treatment response between patients with predicted normal and altered metabolism based on genetic testing. Genetic analysis was performed using two assays based on high-resolution melting PCR (HRM-PCR), which represents an alternative to sequencing for gene analysis. This method is more affordable and easier to integrate into the laboratory workflow in terms of simplicity, turnaround time, and cost.

## 2. Materials and Methods

### 2.1. Patient Cohort and Sample Collection

This study was conducted at the Paediatric Gastroenterology Unit of Regina Margherita Children’s Hospital (Turin, Italy) and included 83 paediatric patients (47 males and 36 females) aged 3–18 years (mean age: 12 years).

Written informed consent was obtained from all participants and/or their legal guardians prior to inclusion in the study. Whole blood samples were collected from all participants. 

### 2.2. DNA Extraction

Genomic DNA was extracted from 300 µL of whole blood that had been collected in EDTA tubes using the Maxwell^®^ 16 LEV Blood DNA Kit (Promega, Madison, WI, USA) and the Maxwell^®^ RSC System (Promega, Madison, WI, USA), according to the manufacturer’s instructions. DNA samples were diluted at 20 ng per microliter and stored at –20 °C until genetic analysis.

### 2.3. TPMT and NUDT15 HRM-PCR Genotyping

Genotyping of *NUDT15* and *TPMT* variants was performed using commercial kits provided by Biomole SRL (Turin, Italy): BM-035 for *NUDT15* and BM-046 for *TPMT*. BM-035 focus on the analysis of NUDT15*1, NUDT15*3 (c.416C>T, rs116855232), NUDT15*6 (c.55_56insGAGTCG, rs869320766); NUDT15*2 (c.55_56insGAGTCG and c.416C>T); and NUDT15*9 (c.50delGAGTCG, rs746071566).

BM-046 can identify the following haplotypes: TPMT*2 (c.238G>C, rs1800462), TPMT*3A (c.460G>A, rs1800460 and c.719A>G, rs1142345), TPMT*3B (c.460G>A, rs1800460), TPMT*3C (c.719A>G, rs1142345).

Both assays are based on high-resolution melting (HRM) analysis, a technique that detects sequence variations by measuring changes in the melting temperature (Tm) of double-stranded DNA. Since each nucleotide sequence has a characteristic Tm, any sequence variation alters the overall melting properties. The *NUDT15* kit uses an intercalating dye that binds non-specifically to double-stranded DNA and generates a fluorescent signal proportional to the amount of double-stranded DNA present, without requiring a sequence-specific probe. In contrast, the *TPMT* kit uses a sequence-specific probe that binds to the target region containing the mutation site, and the fluorescence signal reflects sequence-specific melting profiles. For each sample, 100 ng of genomic DNA were amplified using the amplification mix included in the kit, following the manufacturer’s instructions. Amplifications were performed on a Bio-Rad CFX96 Real-Time PCR System (Hercules, CA, USA). During HRM analysis, the temperature gradually increased, and fluorescence was detected at regular intervals; as the DNA denatured, the fluorescence signal decreased. By analysing the resulting melting curve, the Tm of the amplified sequence was determined.

### 2.4. Sequencing

Following HRM screening of all samples, to confirm the genotyping results, all patients identified as carrying variant genotypes, as well as 10 randomly selected wild-type samples, were verified by Sanger sequencing for the *NUDT15* and *TPMT* variants.

PCR amplification was performed using a 5 µL DNA sample at a concentration of 10 ng/µL, and a 45 µL amplification mix containing GoTaq HotStart polymerase (Promega, Madison, WI, USA), GoTaq Flexi buffer (Promega, Madison, WI, USA) 1.5 mM MgCl_2_, 5 mM dNTPs and primers at a concentration of 1000 nM, as reported in Table 1.

Amplification was performed on a thermocycler at 95 °C for two minutes, followed by 35 cycles of 95 °C for 15 s, 60 °C for 30 s, and 72 °C for 30 s. The PCR products were then purified using the Illustra ExoProStar kit (Sigma-Aldrich, Darmstadt, Germany). One microlitre of alkaline phosphatase and one microlitre of exonuclease I were added to five microlitres of the amplification product, which was then incubated at 37 °C for 15 min and then at 80 °C for 15 min. The sequencing reaction was set up using 1 µL of BigDye Terminator (Thermo Fisher Scientific, Waltham, MA, USA), 2 µL of sequencing buffer, 0.5 µL of purified DNA, and primers at a concentration of 2 µM (forward and reverse, with the sequences reported above). The reaction was run in a thermocycler at 96 °C for 1 min, followed by 28 cycles at 96 °C for 10 s, 50 °C for 5 s, and 60 °C for 4 min. The sequences obtained from the amplification were purified using magnetic beads, and 4 µL of the eluted amplicons were used for sequencing on an Applied Biosystems 3500 Genetic Analyzer (Thermo Fisher Scientific, Waltham, MA, USA).

### 2.5. Statistical Analysis

Associations between genotype groups (wild-type, *TPMT* variants, *NUDT15* variants) and the occurrence of adverse drug reactions were evaluated using Fisher’s exact test (GraphPad Prism, version 7). Odds ratios (OR) with 95% confidence intervals were calculated to estimate the strength of association between genetic variants and adverse events. A *p*-value < 0.05 was considered statistically significant.

## 3. Results

### 3.1. Patients’ Clinical Characteristics

A total of 83 pediatric patients were included in the study. The majority of patients were diagnosed with inflammatory bowel disease, mainly Crohn’s disease and ulcerative colitis. Data on azathioprine clinical response were available for 71 patients. Fourteen patients (16.9%) lacked data on adverse events related to azathioprine; therefore, they were included in the genotype frequency analysis but excluded from the calculation of adverse event rates. Genotyping for *NUDT15* and *TPMT* variants was performed for all patients. The demographic and clinical characteristics of the cohort are summarized in Table 2.

### 3.2. TPMT and NUDT15 Genetic Analysis and Variant Frequencies

All 83 patients were tested for polymorphism in the genetic sequence of the *TPMT* and *NUDT15* enzymes. The observed frequencies were calculated and compared with the expected frequencies according to the CPIC allele frequency table [23]. Two patients showed the deletion delGGAGTC in exon 1 of the *NUDT15* gene on one allele, classifying them as *1/*9 diplotype. Six patients were heterozygous for the G460A and A719G variants in the *TPMT* gene. The HRM-PCR method cannot discriminate whether these mutations are located on the same allele (*1/*3A) or on different alleles (*3B/*3C), and in the table, they have been included alongside the *1/*3A diplotype because their expected frequencies are more comparable (Table 3 and Table 4).

Sequencing results confirmed the genotypes identified by the HRM-PCR method, with 100% concordance.

### 3.3. Clinical Response to Azathioprine

Clinical response data were available for 70 out of 83 patients. Among them, 64 were wild-type for both *NUDT15* and *TPMT*, two carried the *NUDT15* *1/*9 variant, and four carried the TPMT *1/3A variant. A contingency analysis was conducted on patients with available clinical data to evaluate the association between genotypes and the onset of adverse reactions.

In the 64 wild-type patients treated with azathioprine, adverse reactions occurred in four cases (6.25%), with elevated levels of pancreatin enzymes, nausea and vomiting, and other uncorrelated symptoms. Dose escalation was necessary in 28 patients, while in three cases the dose was reduced following clinical remission. In our cohort, six patients showed different *TPMT* and *NUDT15* genotypes associated with dose adjustments and treatment tolerance. Two patients carried the *1/*9 *NUDT15* diplotype with wild-type *TPMT* (*1/*1). Both tolerated standard doses of AZA (1.5–2.27 mg/kg/day) without major adverse effects, yielding *p* = 1 and OR = 0 compared to wild-type, although one required dose escalation due to disease flare. Patients with *TPMT* variant diplotypes (*1/*3A or *3B/*3C) and wild-type *NUDT15* (*1/*1) started AZA at lower initial doses (0.6–1 mg/kg/day). In this group, 2 out of 4 patients (50%) carrying the *TPMT* *1/*3A variant experienced adverse reactions, which was significantly higher compared with wild-type patients (Fisher’s exact test, *p* = 0.0364; OR = 15); results are shown in Table 5.

## 4. Discussion

Personalized healthcare tailors drug dosing to an individual’s genetic profile, improving efficacy, reducing adverse effects, and lowering costs.

Pharmacogenetic data are crucial for therapy selection, especially in paediatric gastrointestinal diseases, where genetic variants affect drug pharmacokinetics and pharmacodynamics [26]. Organizations such as the Clinical Pharmacogenetics Implementation Consortium (CPIC^®^) [27] and the Dutch Pharmacogenetics Working Group (DPWG) provide guidelines for recommended dosage and/or drug choice.

Although no curative therapy exists for chronic autoimmune and inflammatory diseases, several drugs can induce and maintain remission, reducing progression and complications [28,29]. Thiopurines, including azathioprine (AZA) and 6-mercaptopurine (6-MP), are commonly used as steroid-sparing and maintenance therapies in IBD and other autoimmune disorders [30,31]. However, response and toxicity vary markedly; up to 9% of patients are resistant, and adverse reactions occur in up to 28%, often leading to treatment discontinuation [32].

According to the CPIC guidelines, thiopurine dosage should be adjusted according to *TPMT* and *NUDT15* genotypes in order to minimize the risk of haematological toxicity. Standard starting doses of azathioprine (2–3 mg/kg/day) are recommended for normal metabolizers, whereas intermediate metabolizers (an individual who carries one functional allele and one non-functional allele) should receive 30–80% of the standard dose.

For individuals with low or absent *TPMT* activity (An individual homozygous or compound heterozygous for non-functional alleles), the risk of severe, potentially life-threatening myelosuppression is very high. In these cases, guidelines recommend either using alternative non-thiopurine immunosuppressants or drastically reducing the dose (to about 10% of the standard starting dose) with very close haematological monitoring [2]. For individuals who are *NUDT15* normal metabolizers, there is no need to adjust their initial dosage. However, for those who are *NUDT15* intermediate metabolizers, it is advisable to consider reducing the initial dosage to minimize toxicity. For those who are *NUDT15* poor metabolizers, it is recommended to substantially reduce the dosage (for example, to 10 mg/m^2^/day) or to use an alternative agent [2]. In our cohort, patients carrying *TPMT* variant diplotypes *1/*3A and wild-type *NUDT15* (*1/*1) started azathioprine at lower initial doses (0.6–1 mg/kg/day). Two out of four of these patients (50%) developed adverse reactions, supporting the need for genotype-guided dose adjustment. Conversely, patients with wild-type *TPMT* (*1/*1) and the NUDT15*1/*9 diplotype initiated therapy at the standard starting dose, which was subsequently adjusted—either increased or decreased—based on clinical tolerance and hematological monitoring, and were generally able to reach therapeutic doses without major toxicity.

This study analysed genetic variations in thiopurine metabolism genes using two HRM-PCR assays, a sensitive, specific, and cost-effective method. However, it cannot always distinguish certain *TPMT* alleles (e.g., *3A vs. 3B/3C), which may affect metabolizer classification and dose adjustment interpretations.

A total of 83 patients with IBD and other autoimmune diseases had their *TPMT* and *NUDT15* genotypes analysed. Among them, 2 patients (2.4%) carried the *NUDT15* exon 1 deletion (delGGAGTC), classifying them as *1/*9 diplotype carriers, with an expected CPIC frequency of 0.36% in the European population. Regarding *TPMT*, 6 (7.2%) carried heterozygous *TPMT* variants (G460A and A719G), corresponding to the *1/*3A or *3B/*3C diplotypes, with an expected frequency of 6.4% of *1/*3A and 0.003% of *3B/*3C in the European population according to CPIC guidelines. Our expected frequencies are slightly higher in the case of *TPMT* allele *1/*3A than expected and significantly higher in the case of NUDT15 *1/*9. This suggests that the true prevalence of these variants might be underestimated in routine practice, partially because genetic testing for *TPMT* and especially *NUDT15* is still not systematically performed before starting thiopurine therapy. Patients were recruited at a European paediatric hospital, but ethnicity was not recorded and may not be exclusively Caucasian, partly explaining deviations in allele frequencies.

To confirm HRM-PCR results, we sequenced all patients with a variant allele in *TPMT* or *NUDT*, plus 10 randomly selected wild-type patients for each gene, and found 100% concordance with the HRM genotypes. In terms of cost, the sequencing method costs EUR 50 per exon, so EUR 150 for three exons per specimen. In contrast, the BioMole HRM PCR method is expected to cost EUR 25 per specimen; thus, less than one-sixth of the cost of the sequencing method. Sequencing involves complex protocols, specialized equipment, and skilled personnel, with results typically taking several days, whereas the PCR protocol is faster, requiring only extraction and HRM-PCR, with a total turnaround of ~2.5 h.

Nevertheless, Sanger sequencing remains essential in the clinical laboratory, as it is the gold standard for detecting the full range of SNPs and for confirming results when alternative assays show discrepancies or fail. The system described here allows accurate and appropriate genotyping in a clinical laboratory setting, allowing the correction of the thiopurine dose based on the genotyping results of each patient.

Patients carrying the TPMT *1/*3A variant had a markedly higher risk, with 50% showing adverse effects. In contrast, the two patients with the NUDT15 *1/*9 diplotype tolerated standard doses and did not experience any adverse events, although the small sample size limits statistical conclusions. Although our cohort is relatively small, these preliminary findings provide valuable insight into the potential impact of *TPMT* and *NUDT15* variants on thiopurine-related adverse reactions, underscoring the need for larger studies to validate these associations. Genotype alone is not sufficient to predict all adverse events, as other factors—including drug metabolite levels, concomitant therapies, disease activity, and individual patient characteristics—also contribute to treatment response and safety. Therefore, the management of patients undergoing thiopurine therapy should rely on a comprehensive clinical algorithm that integrates several key components: pre-treatment genotyping to guide initial dosing, ongoing monitoring of drug levels and clinical response, and timely therapeutic adjustments including dose modification, treatment interruption, or switching to alternative therapies if needed.

In some clinical settings, *TPMT* enzyme activity can also be directly measured in erythrocytes, providing complementary information to genotyping and further refining dose optimization [33]. It is essential to further promote pharmacogenetics in clinical practice, as this is still a relatively new and rapidly evolving field. Currently, there is a lack of large-scale data regarding the relationship between genotype and thiopurine-related toxicity, the optimal dosing for patients carrying genetic variants, and the true prevalence of these mutations across different populations.

Pharmacogenetic research can benefit from simpler, faster, and more cost-effective approaches than traditional sequencing to facilitate broader implementation in clinical settings. HRM-PCR genotyping, as used in this study, enables accurate and rapid amplification of target gene regions, providing a practical alternative to Sanger sequencing. Consequently, integrating PCR-based genotyping into pharmacogenetic algorithms can accelerate personalized dosing decisions and improve patient management without compromising diagnostic reliability.

## 5. Conclusions

In conclusion, our findings confirm that while pre-treatment pharmacogenetic testing for *TPMT* and *NUDT15* is essential to choose the initial drug dose and reduce the risk of thiopurine toxicity, it must be combined with careful clinical monitoring and flexible dose adjustments to ensure optimal treatment outcomes. Cost-effective, rapid genotyping methods such as HRM-PCR can facilitate broader implementation of personalized algorithms in routine practice, helping to make precision medicine a standard of care in pediatric gastroenterology.

## Figures and Tables

**Table 1:** Primer used for *TPMT* and *NUDT15* sequencing.

*NUDT15* exon 1 primer forward	5′-CAAAGCACAACTGTAAGCGACT-3′
*NUDT15* exon 1 primer reverse	5′-GCAAAGACCTCGCCTGACCCA-3′
*NUDT15* exon 3 primer forward	5′-AGCCAAGCAAATGCAAAGCA-3′
*NUDT15* exon 3 primer reverse	5′-TGGCTGAAAGAGTGGGGGATA-3′
*TPMT* 460 Primer forward	5′-GGACGCTGCTCATCTTCTTAAAG-3′
*TPMT* 460 primer reverse	5′-AGCCTTATAGCCTTACACCCAG-3′
*TPMT* 238 primer forward	5′-CTTTGAAACCCTATGAACCT-3′
*TPMT* 238 primer reverse	5′-CAATTATTTACCCAAATCAAAACAAACC-3′
*TPMT* 719 primer forward	5′-GAATCCCTGATGTCATTCTTCA-3′
*TPMT* 719 primer reverse	5′-CCATTACATTTTCAGGCTTTAG-3′

**Table 2:** Patients’ characteristics: age, sex, diagnosis, and azathioprine response data.

PATIENT	N (%)
Total	83
Age	3–18 [Mean 12]
Males	47 [56.6%]
Females	36 [43.4%]
Underlying pathology	
Ulcerative colitis	25 [30.1%]
Chron’s disease	32 [38.6%]
Autoimmune hepatitis	10 [12%]
Very early onset of inflammatory bowel disease	6 [7.2%]
Indeterminate inflammatory bowel disease	2 [2.4%]
Lymphocytic colitis	1 [1.2%]
Patients with comorbidities	6 [5%]
Ulcerative colitis + sclerosing cholangitis	5 [6%]
Chron’s disease + sclerosing cholangitis	1 [1.2%]
Ulcerative colitis + sclerosing cholangitis + autoimmune hepatitis	1 [1.2%]
Azathioprine clinical response data	
Available	71
Not available	14

**Table 3:** Expected CPIC (Clinical Pharmacogenetics Implementation Consortium) vs. observed frequencies of *TPMT* variant alleles.

*TPMT* Allele	European CPIC Frequency %	Frequency in the Study Cohort %
*1/*1	90.9	92.8
*1/*2	0.4	
*1/*3A	6.5	7.2
*1/*3B	0.5	
*1/*3C	0.9	
*2/*2	0.0	
*2/*3A	0.0	
*2/*3B	0.0	
*2/*3C	0.0	
*3A/*3A	0.1	
*3A/*3B	0.0	
*3A/*3C	0.0	
*3B/*3B	0.0	
*3B/*3C	0.0	
*3C/*3C	0.0	

**Table 4:** Expected CPIC (Clinical Pharmacogenetics Implementation Consortium) vs. observed frequencies of *NUDT15* variant alleles.

*NUDT15* Allele	European CPIC Frequency %	Frequency in the Study Cohort %
*1/*1	98.6	97.6
*1/*2	0.0	
*1/*3	0.4	
*1/*4	0.0	
*1/*5	0.0	
*1/*6	0.6	
*1/*9	0.4	2.4

**Table 5:** Azathioprine dosing and occurrence of hematological, gastrointestinal, and other toxicities in patients with different *TPMT* and *NUDT15* genotypes.

Genotype	Phenotype	Azathioprine Dose	*n*	Any Toxicity *n* (%)	Hematological Toxicity *n*	GI Toxicity *n* (%)	Other Toxicity *n* (%)
TPMT *1/*1; NUDT15 *1/*1	Normal metabolizer	Standard dose 1.5–2.27 mg/kg/day	64	4 (6.25%)	0	3 (4.6%)	1 (1.5%)
TPMT *1/*3A	Intermediate metabolizer	Lower dose 0.6–1 mg/kg/day	4	2 (50%)	0	0	2 (50%)
NUDT15 *1/*9	Possible Intermediate metabolizer	Standard dose 1.5–2.27 mg/kg/day	2	0	0	0	0

## Data Availability

Data will be available upon request.
